# Some Observations on the Preparation and Medicinal Employment of the Ioduret and Hydriodate of Iron

**Published:** 1834-10-01

**Authors:** 


*Some Observations on the Preparation and Medicinal Employment of the Ioduret and Hydriodate of Iron.* By ANTHONY TODD THOMSON, M.D. &c.—London, 1834. 8vo. pp. 64.

IN a former Number of our Journal, (vol. ii. p. 82,) we extracted Dr. A. T. Thomson's recommendation of the ioduret of iron, from his paper on *Stimulants*, in the Cyclopædia of Practical Medicine. His further experience has now produced a work which, though small, is interesting, and from which we shall therefore make some large extracts, for the gratification of our readers.

As these new preparations are not yet kept, we believe, in the majority of shops, practitioners (especially those residing in the country,) will find the following directions very useful; and the more so, as the solution of the hydriodate of iron is one of those delicate medicines whose qualities are as mutable as those of the atmosphere which decomposes them; while this new compound is dangerous when its composition is dubious: so that, to use a trite simile, it must be put away, like Cæsar's wife, unless it is not only pure, but above suspicion.

" *Precautions necessary in the Preparation of the Ioduret and the Hydriodate.* One part of the iron wire should be rubbed in a porcelain or a Wedgwood's mortar, with about three or four parts of iodine, gradually adding distilled water, until fifteen parts of the fluid shall have been used: the whole is then to be introduced into a Florence flask, with an additional portion of iron wire, and of distilled water. This excess of iron is a matter of indifference in the preparation of the hydriodate; and, in that of the ioduret, it is necessary for preserving the combination from decomposition, during the evaporation of the solution. These materials are next to be boiled together, until the fluid acquire a pale greenish colour, when it should be filtered. This solution contains a hydriodate of the protoxide of iron; and, if the exact quantity of the iodine be previously ascertained, so as to enable us to procure the solution of a definite strength, it may be kept in this state for medicinal use. In general, however, the solution is evaporated to dryness; and for this purpose it may be poured into a clean flask, containing a piece of iron wire sufficiently long to reach from the bottom to the surface of the fluid, and the boiling should be continued until the bulk of the solution be reduced to one third. It must then be filtered; after which the evaporation should be continued to dryness. It is necessary to break the flask as soon as the mass is cold, in order to obtain the solid ioduret, which should be immediately transferred to a dry bottle, fitted with an accurately ground stopper. The bottle should not hold more than two ounces of the preparation; for, when it is large and not full, the ioduret deliquesces nearly as rapidly as when it is exposed to the free action of the atmosphere. When the flask is broken and the ioduret bottled before the mass is cold, deliquescence also takes place: a peroxide of the metal is formed, and iodine is evolved.

" 3. *Precautions necessary for preserving both Preparations.* The ioduret requires to be well secured from the influence of the atmosphere; both on account of its deliquescent property, the rapid oxidizement which the iron undergoes when deliquescence occurs, and the consequent decomposition which takes place. It is important to prevent this state of things, as the peroxide of iron is inert as a medicinal agent; whilst the free iodine, extricated during its formation, alters altogether the virtues of the medicine. This partial decomposition of the ioduret is rendered immediately apparent, on dissolving it in twenty times its weight of distilled water and filtering: instead of a permanent, clear, very pale greenish yellow, we obtain an ochre-coloured or brown solution, which soon becomes turbid, and gradually deposits an ochre-coloured completely insoluble precipitate. Much of the iodure usually prepared both by many chemists and druggists, and also general practitioners, is of this description; and to this we may refer some of the disappointment and discrepancy of opinion of different practitioners respecting the operation of the medicine in similar cases. Even when the ioduret has been carefully prepared, and is good of its kind, it often contains a little free iodine: but it is chiefly owing to the carelessness of assistants and apprentices, in compounding prescriptions, by frequently exposing the ioduret to the air, that its properties, and consequently its medicinal powers, are impaired: thence, it is preferable to keep it in solution, or in the state of the hydriodate.

"If the solution be prepared with a definite quantity of iodine, as already described, it will keep without changing its characters: but, as it is usually made by dissolving the ioduret in distilled water, it requires to be rendered neutral by the following means. Introduce into a flask the solution of any given strength, and place in it two or three doubles of clean, soft iron wire, sufficiently long to extend to the surface of the fluid; boil it for a few minutes, and then leave it at rest until the solution become clear, after which it may be either decanted off from the precipitate which forms, or filtered: no further change takes place in a solution thus treated if it be kept in a blackened or a green bottle, however long it may be preserved. In this process, the wire affords iron to saturate any free iodine present in the solution, or that may have been extricated by the formation of the peroxide of iron in the ioduret; and a perfectly neutral solution being thus obtained, by the immediate conversion of the new-formed ioduret into the hydriodate of the protoxide, no subsequent change takes place as long as the solution is kept secluded from the light. It is not easy to explain this influence of light in decomposing the solution of the hydriodate of iron; but several other of the metallic hydriodates are affected in the same manner by light. The best proportions for forming the medicinal solution are three grains of the dry solid ioduret to each fluid drachm of distilled water. If the water be not either distilled, or filtered rain water, perfectly free from foreign ingredients, particularly if it contain any earthy or saline carbonates, decomposition instantly takes place, iodine is extricated, and a carbonate of iron, which rapidly passes into the state of the peroxide of that metal, is precipitated." (P. 7.)

It is impossible to read these minute and scientific precautions, without reflecting on the ordinary coarseness of pharmaceutical manipulation, and the total disregard of accuracy, which prevail not only among private practitioners and in druggists' shops, but even in those institutions where publicity, and the presence of physicians, might seem to ensure tolerable correctness. Those who have seen the singular makeshifts and odd surrogates of dispensary practice might hesitate before they added the hydriodate of iron to their scanty pharmacopœias: as Goldsmith says,

> " Such dainties to them, it would look like a flirt,
> Like sending 'em ruffles, when wanting a shirt."

Our author thinks that the solution is of a strength best suited to medicinal use, when it contains three grains of the hydriodate in each fluid drachm. When it is of this strength, any shade of brown indicates the presence of free iodine, " which is also demonstrated by the blue colour communicated to thin, cold mucilage of starch; for, when the solution is perfectly neutral, no blue colour is evolved." (P. 12.) The solution of the hydriodate is decomposed by chlorine, the mineral acids, arsenious acid, meconic acid, gallic acid and tannin, the pure alkalies, pure cinchonia or quinia, the carbonates of alkalies, sulphate of copper, nitrate of lead, the proto- and per-nitrate of mercury, nitrate of silver, arsenite of potass, hydrosulphate of potass, hydrosulphate of ammonia, acetate of lead, oxalate of ammonia, and hydrocyanate of potass; by the infusions, decoctions, and tinctures of galls, oak bark, uva ursi, pyrola umbellata, cloves, roses, cinchona, cusparia, and all vegetable substances containing gallic acid or tannin, the infusions of foxglove, belladonna, hyoscyamus, and tobacco; and, lastly, by all vegetable infusions and decoctions containing fecula, for they afford a blue precipitate (the iodide of amidine,) when there is any free iodine in the solution.

" On the other hand, it may be ordered in combination with all the saline sulphates, nitrates, muriates, chlorates, and phosphates: with bitter vegetable infusions and decoctions containing neither tannin nor gallic acid, namely, simple infusion of orange peel (the tannin of the cloves in the compound infusion of the London and Dublin Pharmacopœias causes a black precipitate), buchu, gentian, quassia, senega root, white hellebore, dulcamara, &c." (P. 16.)

We now come to its practical use. Our author gives thirteen cases in which the hydriodate of iron has been employed with success by himself and other physicians. He divides them into four classes; the first illustrates the beneficial effects of the remedy in scrofula, the second in chlorosis, the third in carcinoma, and the fourth in syphilitic cachexia and eruptions. The first case is that of a young lady, æt. fourteen, who was sent home from school on account of swellings of the cervical glands, accompanied by cough and fever.

"I first saw her on the eighth of August, 1833. There was a chain of indurated glands extending from below the middle of the lower jaw to behind the ear on the right side, two of them in a state of active suppuration; whilst one tumour, nearly the size of half of an egg, rose, free from any external redness, directly under the angle of the left jaw. Her general health was greatly disordered; the pulse was small, but sharp and brisk; the tongue was redder than natural, and covered, at the back part, with a slimy fur; the bowels were relaxed; and the catamenia, which had occurred, for the first time, a month after the swellings began, had not again appeared. From the sound of the voice, I suspected that the tonsils were affected; and, on examination, I found that they were inflamed and considerably enlarged. My patient complained of almost continued headachs; great prostration of strength; and, since the cough had supervened, she was still more weakened than before by morning perspirations. Under these circumstances, my first object was to moderate the inflammatory action, to allay the irritability of the digestive organs, and to improve the secretions: for which purpose, I ordered her to be cupped between the shoulders, and to take the following medicines:—℞. Hydrargyri cum Creta, gr. xii.; Pulveris Ipecacuanhæ compositi, gr. x. M. ut fiat Pulvis hora somni quotidie sumendus.—℞. Olivæ Olei, f ℥iss. Acaciæ Gummi pulveris, ℥ss.; Hydrocyanici Acidi, m. iii.; Aquæ Distillatæ, f ℥viiiss.; Sacchari albi, gr. x. M. ut fiat haustus ter die sumendus.

"Her diet was ordered to be light, but nutritious; to consist chiefly of preparations of milk, morning and evening, with a moderate allowance of mutton or poultry once a day, and well-boiled vegetables. Wine and all fermented liquors were interdicted. Friction was directed to be applied along the course of the spine, and over the abdomen; and exercise in an open carriage was recommended to be taken daily, except when the wind was from the east or the north.

"August 9th. One of the tumours was opened today, and syringed with a solution of sulphate of zinc. The powder and draughts were ordered to be continued.

"August 17th. The same plan of treatment has been pursued since the 9th instant, with the addition of the application of four leeches two days ago, on account of the tumour on the left side becoming tender; the occasional interposition of a saline purgative has been also necessary. The condition of the digestive organs is much improved; the tongue is nearly natural; and the cough has ceased. The enlarged glands, however, are nearly in the same state as when I first saw the patient, except that the other suppurating tumour has been opened. As the ulcer which succeeded the opening of the first tumour is very irritable and painful, let a hemlock and foxglove poultice be laid over it.—Pergat in usu pulveris, addendo Aloës pulveris, gr. iv.—℞. Ferri Iodureti, gr. iij.; Aquæ distillatæ, f ℥xi.; Aurantii Tincturæ, f ℥i. M. ut fiat haustus ter die sumendus.—℞. Iodinii, ʒss.; Potassæ Hydriodatis, ʒiss.; Adipis purificati, ℥i. Tere diligenter ut fiat unguentum; pauxillum tumoribus cervicis, ope fricationis, mane et vespertino, quotidie applicandum.

"In ten days the mouth became tender, consequently the powder was discontinued. The bowels were then regulated by the occasional administration of a five-grain aloetic pill; and the other parts of the treatment were pursued, with little variation, for two months, during which time the catamenia returned, and the improvement in health and strength was progressive and striking. The tumour on the left side suppurated, but all the ulcers rapidly cicatrized, while the swellings gradually softened and dispersed; and on the 20th of October my patient returned to school in a much improved state of health." (P. 24.)

There are three cases of chlorosis, (two of them communicated by Dr. C. J. R. Williams,) in which the hydriodate was employed with great advantage. Our author was at first unwilling to give up his previous method of treating this disease, with sulphate of iron in combination with the pill of aloes and myrrh; but the first case, in which the chlorosis was ingrafted upon a scrofulous habit, having resisted these remedies, he tried the hydriodate of iron with a success which induced him to administer it to other chlorotic patients.

"Mary ——, nineteen years of age, a patient in the Dispensary of the University of London, has been gradually declining in health for the last five months. She now complains of great languor; extreme depression of spirits; defect of appetite, a torpid state of the bowels; recurrent headach, coming on generally twice in the twenty-four hours; embarrassed breathing; and a disposition to hysteria, denoted by the globus hystericus, and great nervousness. The whole skin is of a pale sallow hue, the lips nearly white, and the expression of the countenance languid and anxious. The menstrual discharge has not appeared for two months; and was previously extremely scanty and pale coloured. She says that she nevertheless sleeps soundly, but is unrefreshed in the morning; and, long before the day is half spent, her ankles and feet swell to a degree which prevents her from wearing her shoes. The bowels are irregular; most commonly costive, but occasionally they are purged. She is a straw-plaiter; and I am inclined to ascribe her disease to her sedentary habits, a strumous diathesis, and a natural delicacy of constitution. The following medicines were prescribed. —℞. Pilulæ Submuriatis Hydrargyri compositæ, gr. xx.; Aloës Soccotrini Extracti, ℈iv.; Mucilaginis Acaciæ, q. s. ut fiant pilulæ viginti æquales. Sumantur i horâ somni quotidie. ℞. Ferri Iodureti, ʒi.; Aquæ Distillatæ, f℥iv. Solve et cola. Sumatur cochl. med. i ter quotidie.

"The patient was ordered to take as much exercise in the open air as her strength and circumstances would admit; and to let her diet be mild, but as nutritious as she could procure.

"She pursued this plan for a month, gradually increasing the dose of the solution to two spoonfuls, equal to four grains of the ioduret: no other medicines, with the exception of an occasional purgative of salts and senna, were prescribed. In less than a week both the headachs and languor disappeared; her lips and general complexion rapidly acquired a healthy hue; the catamenia returned in the middle of the fifth week: and, when she left the Dispensary, she was apparently in as good health and spirits as could be desired." (P. 41.)

It does not appear, from Dr. A. T. Thomson's account of this case, at what period the ordinary treatment had been adopted without success, but we conjecture that it must have been resorted to by some other practitioner, during the five months that the patient's health was declining.

There is only one case of carcinoma, but that is a satisfactory one. The patient was a married woman, forty-six years old, who had never borne children, and in whom the catamenia had finally disappeared. One case indeed can prove but little, as our author himself states with laudable candour.

"I am fully aware, in offering this case as an instance of the advantage of the use of the hydriodate of iron in carcinoma, that little reliance can be placed on a solitary case; but I am desirous that it should be tried by others. In none of the numerous diseases to which humanity is liable, is a fatal termination so probable as in cancer: at the same time, whilst the deposition of the carcinomatous matter is going on, and the disease is yet merely local, there is no reason for refraining from trying means that seem to promise a favorable issue." (P. 53.)

The last division contains four cases. In the first, (communicated by Sir David Barry,) the patient, who denied that he had ever suffered from the venereal disease, was affected with chronic ulceration of the posterior fauces. After a variety of treatment had been employed without advantage, the ioduret of iron was tried, and the disease was cured in a month. In this case, however, a relapse soon took place.

"On the 8th of February, 1834, Collins presented himself again, with a slight return of ulceration in the throat. He was again put upon the use of the ioduret of iron, and is now [February 20th] better." The other three were still more satisfactory, as the cure appears to have been permanent. We must content ourselves with one only.

"S— D—, a young gentleman, nineteen years of age, has suffered, during several years, from a leprous affection, which has a very suspicious copper colour, and is attended with sore throat, and febrile symptoms. I have not been able to trace this eruption to any syphilitic disease, although I strongly suspect that such has been its origin. It is always most severe in summer; abating considerably in violence during winter. The skin in many places is scarred with the cicatrices of sores; but I can get no satisfactory information respecting them.

"This gentleman was recommended to try an alterative course of Plummer's pill and sarsaparilla, which he continued for three months without any decided benefit being obtained; he was therefore put upon a course of the ioduret of iron, which he took in doses of four grains three times a day, and employed a moderately stimulating gargle. He improved rapidly, and in four weeks was perfectly well." (P. 64.)

Our author concludes by observing, that he has used the hydriodate with advantage in many cases of atonic dyspepsia.

The present pamphlet, like Dr. Thomson's other works, is replete with valuable matter, and has all the diagnostic marks by which we distinguish the writings of the genuine practical physician from those of the dreamer or the impostor. The only serious defect we can find in it, is one with which we rarely reproach medical authors—it is too short. We trust that the experience of a few more years will enable Dr. Thomson to amend this fault, and that he will have had the glory of adding to our Pharmacopœia a remedy combining the virtues of two such powerful agents as iron and iodine.

